# Tocilizumab and Rituximab in Systemic Sclerosis: A Real-Life Retrospective Observational Study Across Different Clinical Phenotypes

**DOI:** 10.3390/jpm16040186

**Published:** 2026-03-30

**Authors:** Silvia Cavalli, Maria Rosa Pellico, Giorgia Trignani, Manuel Sette, Claudia Iannone, Roberto Caporali, Nicoletta Del Papa

**Affiliations:** 1 Department of Clinical Sciences and Community Health, University of Milan, 20122 Milan, Italy; silvia.cavalli@ospedaleniguarda.it (S.C.); mariarosa.pellico@unimi.it (M.R.P.); giorgia.trignani@unimi.it (G.T.); doc.manuelsette@gmail.com (M.S.); claudia.iannone@unimi.it (C.I.); roberto.caporali@unimi.it (R.C.); 2 Scleroderma Clinic, Clinical Rheumatology Unit, ASST Gateano Pini-CTO, 20122 Milan, Italy

**Keywords:** systemic sclerosis, disease activity, biologic agents, tocilizumab, rituximab

## Abstract

**Objectives**: To describe the efficacy and safety of tocilizumab (TCZ) and rituximab (RTX) in real-world patients with systemic sclerosis (SSc), across different clinical phenotypes and lines of therapy, evaluating both global clinical outcomes and lung function. **Methods:** SSc patients treated with TCZ (*n* = 27) or RTX (*n* = 23) were retrospectively followed for 12–24 months. Clinical measures, including modified Rodnan Skin Score (mRSS), C-reactive protein (CRP), and revised EUSTAR activity index 2017 (RAI), as well as spirometric parameters, were recorded at baseline and 6, 12, and 24 months. Statistical methods for repeated measures were applied to investigate outcome trends. Given the baseline differences, between-group comparisons were considered exploratory. **Results:** RTX was used earlier in disease course, while TCZ was mainly used as a rescue therapy. In both groups, mRSS, CRP levels and RAI significantly decreased over time. RTX-treated patients showed a greater absolute mRSS improvement, in line with higher baseline skin scores. No treatment discontinuations due to adverse events occurred in either group; one death and one discontinuation due to inefficacy were observed in the TCZ group. Among SSc-ILD patients, FVC% showed a modest decline in both groups, while DLCO% remained overall stable, and only a few patients met the OMERACT criteria for functional progression. **Conclusions:** In this real-world, single-center cohort of SSc patients, both agents were associated with a positive impact on disease activity, with a low rate of lung progression and with favorable safety profiles. Owing to substantial baseline imbalances and confounding by indication, between-group comparisons do not allow firm conclusions on comparative effectiveness. Overall, these data support the use of both agents in different clinical scenarios.

## 1. Introduction

Systemic sclerosis (SSc) is a rare autoimmune disease driven by inflammation, vasculopathy, and fibrosis, affecting many different organs, primarily the skin, lungs, gastrointestinal tract, musculoskeletal system and heart.

Currently, interstitial lung disease (ILD) represents the leading cause of death in SSc patients, affecting approximately 50–65% of cases. The predominant pattern is non-specific interstitial pneumonia (NSIP), with considerable variability in both functional and radiographic progression [1,2,3].

SSc treatment remains challenging, both for the complexity of the disease itself and for the lack of robust evidence from randomized controlled trials. For autoimmune features of the disease, current management typically includes conventional synthetic immunosuppressants such as cyclophosphamide (CYC), methotrexate (MTX) or mycophenolate mofetil (MMF), tailored according to disease severity and organ involvement. Significant advances have emerged with biologic therapies, namely rituximab (RTX), an anti-CD20 monoclonal antibody, and, more recently, tocilizumab (TCZ), an anti-IL6 receptor monoclonal antibody showing efficacy in skin and lung involvement [4,5,6,7]. Additionally, for progressive fibrosing SSc-ILD, the approval of nintedanib (NNT), an antifibrotic drug with established use in idiopathic pulmonary fibrosis, represents another important therapeutic milestone [8,9]. These advances are reflected in the most recent EULAR therapeutic recommendations for SSc, published in 2023, which include both RTX and TCZ for skin and lung involvement, and NNT for this latter indication [10].

Although the advent of biologic therapies is opening promising new avenues in the treatment of many connective tissue diseases (CTDs), SSc still lacks real disease-modifying treatments, with the potential exception of autologous hematopoietic stem cell transplantation (AHSCT) [10]. Although randomized controlled trials (RCTs) have demonstrated efficacy of CD20 and IL6 pathway inhibition compared to placebo in SSc patients, real-world data—especially for TCZ—remain limited. Moreover, direct comparative studies between different therapeutic mechanisms are scarce.

Recent retrospective studies conducted on real-world SSc cohorts have addressed this topic, focusing on interstitial lung disease: Goldman et al. compared TCZ with RTX on 127 SSc-ILD patients, while Yan et al. conducted an analysis on the EUSTAR cohort comparing TCZ with other immunosuppressive therapies (including cyclophosphamide, methotrexate, azathioprine, mycophenolate mofetil, abatacept, and rituximab): no significant differences in FVC trends were underlined in either study, while no other extra-pulmonary clinical outcome was analyzed [11,12].

In clinical practice, TCZ and RTX are often used in different clinical scenarios and at different stages of disease progression, reflecting distinct phenotypes, therapeutic sequences, and treatment indications.

This study aims to describe the real-world effectiveness and safety of TCZ and RTX in SSc patients, evaluating both lung function and global clinical outcomes across different clinical phenotypes and treatment lines. Given substantial baseline differences between patients receiving each agent, comparisons between the two groups should be interpreted as exploratory observations rather than definitive comparative assessments.

## 2. Materials and Methods

Patients diagnosed with SSc and fulfilling the 2013 EULAR/ACR classification criteria, aged > 18 years old and treated for at least 12 months with either TCZ (RoActemra 20 mg/mL, Tokio, Japan, 162 mg via weekly subcutaneous injections) or RTX (Rixathon 500 mg/50 mL, Holzkirchen, Germany, 1000 mg at weeks 0 and 2, intravenously, every 6 months) were enrolled [13], were included. The initiation of the drug was considered as the baseline and the inclusion period was between 2018 and 2021. Exclusion criteria were age < 18 years old at the time of enrollment, treatment duration < 1 year, and follow-up < 18 months. The minimum treatment duration was set at 12 months, as at the time of data analysis only 21 (77.8%) TCZ-treated patients had completed the full follow-up period. The study was conducted in accordance with the Declaration of Helsinki and approved by the Institutional Ethics Committee (ID study 3339, study number 6549).

Patients were followed from the introduction of the drug (T0) and reassessed at 6 months (T6), 12 months (T12) and 24 months (T24). Data were collected from electronic health records. In detail, demographic and clinical data, autoantibody status and concomitant immunosuppressive therapies were registered for each patient. Modified Rodnan Skin Score (mRSS), C-reactive protein (CRP) levels and the revised EUSTAR activity index 2017 (RAI) were collected at each time point [14]. CRP values were not recorded if an infection or other confounding condition was present: in such cases, another measurement within two weeks of the predefined time point was selected. Pulmonary function tests (PFTs) were performed according to standard international recommendations using calibrated spirometry and single-breath DLCO measurement. In detail, forced vital capacity (FVC%) and diffusion capacity for carbon monoxide (DLCO%) were recorded as percentage of predicted values based on reference equations adjusted for age, sex, height, and ethnicity. Results of PFTs were collected at T0, T6, T12, and T24 in patients with interstitial lung disease.

### Statistical Analysis

Statistical analysis was performed using SPSS v.29.0.1.0 and R v.4.4.1. Normality of the variables was assessed through Shapiro–Wilk test. Between-group comparisons were conducted through T-test or Mann–Whitney U test for continuous variables and chi-square test or Fisher’s exact test for categorical variables, as appropriate.

For clinical variables (mRSS, CRP and RAI), within-group changes over time were assessed using Friedman test followed by Wilcoxon signed-rank test for pairwise comparisons. A Bonferroni correction was applied to account for multiple testing. Between-group differences in changes over time were explored using Mann–Whitney U test. Given the observational design and relevant baseline imbalances between the TCZ and RTX cohorts, these between-group comparisons were considered exploratory and were not intended to provide definitive evidence of comparative effectiveness. The probability of cutaneous improvement (5 points or 25% reduction in mRSS) and clinical remission (RAI < 2.5) was assessed using Kaplan–Meier curves and the Breslow test [14].

Over time, changes in spirometric values (FVC% and DLCO%) were analyzed using a linear mixed model for repeated measures adjusted for disease duration. As for clinical remission and cutaneous improvement, Kaplan–Meier curves and Breslow test were used to evaluate the probability of spirometric progression, applying the OMERACT definition for CTD-ILD functional progression, namely a >10% absolute decrease in FVC% or >5% and <10% in FVC% with a concomitant absolute decrease > 15% in DLCO% [15].

Missing data were handled through the mixed model framework, which incorporates all available data under the assumption of missing at random (MAR), and by imputation using linear interpolation, as appropriate. Missing data accounted for <10% per each variable. No additional adjustment methods such as propensity score matching or weighting were applied; therefore, residual confounding by indication cannot be excluded.

## 3. Results

### 3.1. Cohort Characteristics

Patients’ main characteristics are shown in Table 1 and in Supplementary Table S1.

In detail, 27 SSc patients received TCZ for at least 12 months. The most common reason for drug introduction was ILD progression (*n* = 15, 55.60%), followed by arthritis (*n* = 6, 22.22%), cutaneous activity (*n* = 3, 11.11%) and myocarditis defined as elevated cardiac troponin I together with MRI evidence of myocardial edema (*n* = 3, 11.11%). Sixteen patients (59.26%) received a concomitant conventional immunosuppressant (MTX in 11 and MMF in five patients), whilst seven patients (25.92%) were already in antifibrotic treatment with NNT at the time of TCZ introduction. For the majority of the patients (*n* = 11, 40.74%), TCZ was the third line of treatment, with a median number of previous immunosuppressants (IS) of two (IQR 2). At baseline, 21 patients (77.78%) had an active disease (RAI > 2.5) and 25 patients (92.60%) were receiving a low–medium dosage of steroid, tapered during the observational period.

In the RTX group, 23 patients receiving the drug for at least 12 months were enrolled. The main indications for RTX introduction were cutaneous (*n* = 13, 56.52%) and ILD progression (*n* = 10, 43.48%). Nineteen patients (82.61%) received a concomitant conventional immunosuppressant (MTX in 12 and MMF in seven patients), whilst no patient was in antifibrotic treatment at the time of recruitment; RTX was the second therapeutic line for 15 (65.22%) patients (median number of previous IS = 1, IQR = 1). At baseline, 21 RTX-treated patients (91.30%) had an active disease and were taking a low–medium steroid dosage, tapered during the follow-up.

As shown in Table 1, the main differences between the two groups were disease duration, higher in the TCZ group (TCZ: median time = 98 months, IQR = 95.50; RTX: median time = 37 months, IQR = 20.50; *p* < 0.001), and baseline mRSS, higher in the RTX group (TCZ: median = 3, IQR = 8; RTX: median = 14, IQR = 10; *p* < 0.001). Across SSc-ILD patients (TCZ: *n* = 19, 70.37%; RTX: *n* = 18, 78.26%), DLCO% was lower in the TCZ group (TCZ: mean = 41, SD = 24.00; RTX: mean = 56, SD = 20.91; *p*: 0.024). Overall, TCZ was preferentially prescribed to patients with more longstanding disease, more impaired gas exchange and more frequent use of antifibrotic therapy, whereas RTX was more often used earlier in the disease course and in patients with higher skin scores, reflecting different treatment indications and clinical phenotypes.

### 3.2. Clinical Outcomes: Trends and Comparison Between Groups

**Figure 1:**
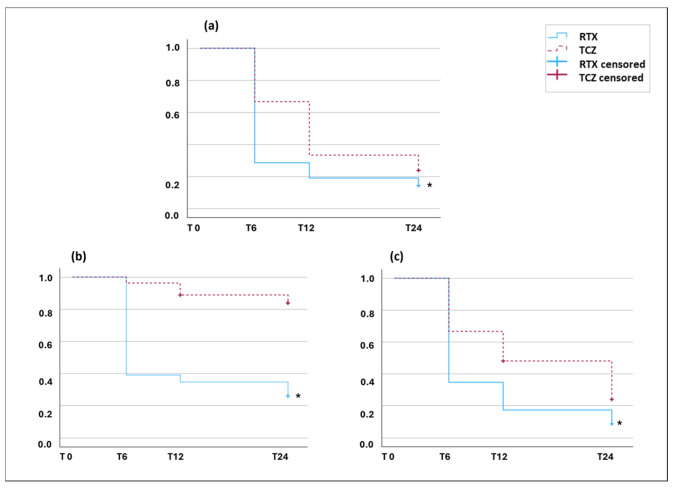
Survival curves of the two treatment groups for predefined cut-offs. TCZ: red line, RTX: blue line. (**a**) Decrease in RAI under 2.5, cohort with a baseline RAI > 2.5 (TCZ: *n* = 21, RTX: *n* = 21); (**b**,**c**) decrease in mRSS > 5 points (**b**) and >25% of the previous recorded value (**c**), whole cohort (TCZ: *n* = 27, RTX: *n* = 23). Breslow test, 1 degree of freedom. (**a**) = Chi: 4.469, * *p*: 0.035; (**b**) = Chi: 19.825, * *p* < 0.001; (**c**) = Chi: 5.948, * *p*: 0.015.

Changes in clinical parameters over time of TCZ- and RTX-treated patients were analyzed and are displayed in Table 2. In detail, there was a statistically significant decrease in mRSS, CRP levels and RAI at each time point with respect to T0 in both groups, with the only exception of CRP levels missing statistical significance at T24 in the RTX group. When over time variations were compared between groups (Supplementary Table S2), RTX-treated patients showed a more marked reduction in mRSS at each time point and in RAI at T6, whereas TCZ-treated patients displayed a more pronounced CRP reduction at T24. Lastly, survival curves for clinical remission and cutaneous improvement were performed: the RTX group showed a higher probability of achieving clinical remission (TCZ: 16 events, RTX: 18 events; *p*: 0.035) (Figure 1a) and mRSS reduction, both in terms of absolute decline (TCZ: four events, RTX: 17 events; *p* < 0.001) (Figure 1b) and percentage decline (TCZ: 18 events, RTX: 21 events; *p*: 0.015) (Figure 1c).

### 3.3. Spirometric Trends and Pulmonary Progression: Analysis and Comparison Between the Treatment Groups

**Figure 2:**
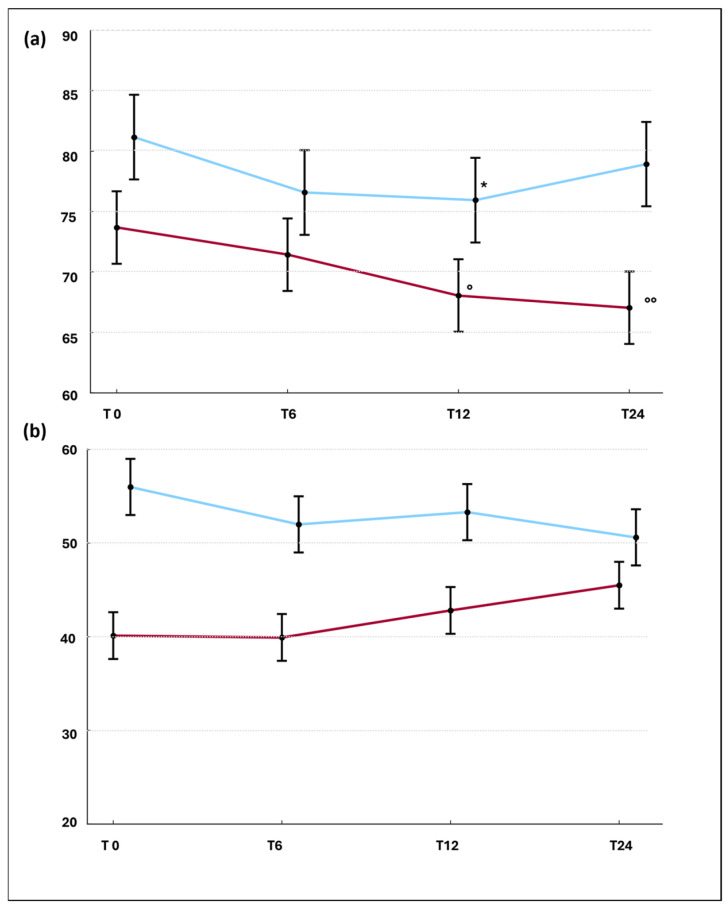
Over time changes in FVC% (**a**) and DLCO% (**b**) in the two treatment groups. TCZ-treated patients: red line, RTX-treated patients: blue line, mean (points) and standard deviations (vertical lines); analysis conducted only on SSc-ILD patients (TCZ: *n* = 19 and RTX: *n* = 18). Linear mixed model for repeated measures. ° TCZ vs. T0 = β: −5.28, *p*: 0.019; °° TCZ vs. T0 = β: −6.47, *p*: 0.04; * RTX vs. T0 = β: −5.22, *p*: 0.0563.

A linear mixed model adjusted for disease duration was used to analyze over time changes in FVC% and DLCO% in the SSc-ILD group of patients treated with TCZ (*n* = 19) and with RTX (*n* = 18). The results are shown in Figure 2a,b, respectively. In detail, TCZ-treated patients showed a significant decrease in FVC% both at T12 (β: −5.28, *p* = 0.019) and at T24 (β: −6.47, *p* = 0.04) with respect to T0, while, within RTX-treated patients, FVC% displayed a decrease in borderline significance at T12 compared to T0 (β: −5.22, *p* = 0.056). In both groups, DLCO% remained stable throughout the follow-up period.

**Figure 3:**
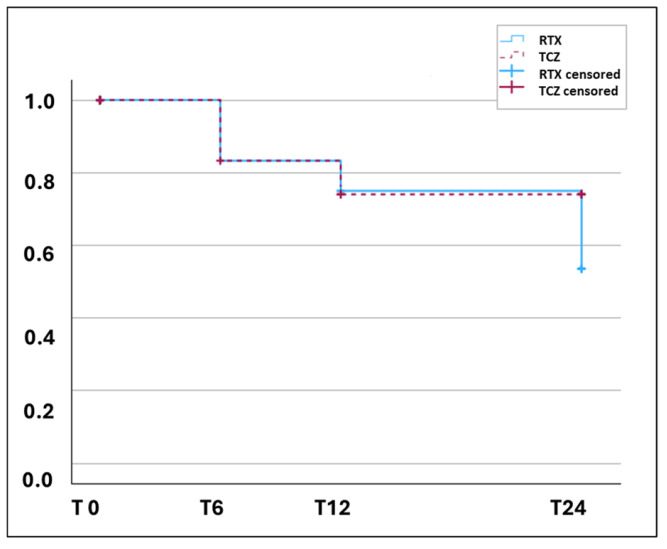
Survival curves of the two treatment groups for predefined cut-offs. TCZ: red line, RTX: blue line. Spirometric progression (OMERACT definition), cohort of SSc-ILD patients (TCZ: *n* = 19, RTX: *n* = 18).

In Figure 3, survival curves for spirometric progression according to OMERACT are shown. In detail, within SSc-ILD patients, only three events in the TCZ group and five in the RTX group were recorded during the whole observational period, without differences between the two groups. Moreover, at the end of the follow-up period, only one patient in the TCZ group (5% of patients not already in antifibrotic treatment at T0) and no patient in the RTX group had added NNT to the treatment.

### 3.4. Prognosis and Safety

The entire follow-up was completed by 21 TCZ-treated patients (77.78%) and 23 RTX-treated patients (100%). At the end of the follow-up period, one death, occurring 9 months after the drug introduction, was recorded in TCZ-treated patients: the event was due to congestive heart failure, with no evidence of a causal relationship with TCZ mechanism of action. Additionally, one patient discontinued TCZ during the observational time due to drug failure, 18 months after its introduction, and was later addressed to AHSCT due to the aggressiveness of the disease. No patient deceased or discontinued the treatment in the RTX group during the whole follow-up period. Overall, no treatment discontinuations due to adverse events were observed in either group over 24 months, supporting a favorable safety profile for both TCZ and RTX in this real-world cohort.

## 4. Discussion

Our retrospective study aimed to evaluate the efficacy of the recently approved anti-IL6 receptor monoclonal antibody tocilizumab and of rituximab—a cornerstone in SSc treatment—in real-life SSc patients, more complex and heterogeneous than most patients enrolled in RCTs [16]. Our study should be interpreted as a descriptive comparison of two treatment strategies in different clinical contexts rather than a head-to-head efficacy analysis. In our particular setting, both RTX and TCZ showed a positive and rapid impact on cutaneous and global disease activity, while lung functional trends were more difficult to interpret, with a modest decline in FVC% and relative stability of DLCO% in both treatment cohorts.

Evidence regarding the efficacy of TCZ in SSc patients derives from two randomized placebo-controlled studies: FaSScinate (phase II) and FocuSSced (phase III) [6,7]. These trials included SSc patients with disease duration of less than five years and elevated inflammatory markers at baseline. Although neither study met its primary endpoint of skin improvement, the TCZ group showed a trend toward mRSS reduction. Concerning lung involvement, both FaSScinate and FocuSSced showed a smaller decline in FVC in TCZ-treated patients compared to placebo. Notably, both trials excluded background immunosuppressive therapy, particularly conventional synthetic immunosuppressants [6,7]. Based on these findings, the Food and Drug Administration (FDA) approved TCZ for the treatment of SSc. Subsequently, EULAR included it within its therapeutic recommendations, both for pulmonary and cutaneous disease, positioning it as an advanced option for the latter indication [10].

RTX, a monoclonal antibody targeting CD20, is widely used for SSc treatment, either alone or in combination with conventional synthetic immunosuppressants. It is typically reserved for patients with aggressive cutaneous involvement or severe interstitial lung disease. Several studies support RTX efficacy in this patient subgroup, particularly two pivotal randomized placebo-controlled trials: the Japanese DESIRES study and the Leiden trial [4,5]. Regarding skin involvement, both trials demonstrated RTX superiority over placebo in achieving mRSS reduction. With respect to ILD, results were mixed: the DESIRES showed improved FVC values at week 24 in the RTX group compared to placebo, while the Leiden trial failed to show any difference in terms of spirometric or radiographic progression between the two groups [4,17]. Additionally, two trials evaluated the efficacy of RTX compared to CYC in SSc-ILD patients, namely the RECITAL basket trial—which included 37 SSc patients—and an open-label RCT by Sircar et al. Both studies favored RTX treatment, also underlying a better safety profile [5,18].

Despite growing evidence supporting both TCZ and RTX in SSc treatment, real-world data remain limited, especially for patients with advanced or longstanding disease. Indeed, the current literature mostly derives from trials conducted in highly selected SSc cohorts, typically including early-stage SSc patients while excluding those with longstanding disease or advanced cardiopulmonary involvement. In contrast, patients in real-world settings often present with a broader spectrum of phenotypes, especially those with longstanding, multi-refractory SSc. These cases are often characterized by minimal cutaneous activity due to progression into the atrophic phase, yet exhibit varying degrees of activity in other organ systems. Moreover, RCTs often assess treatment efficacy based on single-organ outcomes, without using composite disease activity scores, and are limited by inconsistent definitions of SSc and SSc-ILD progression, despite recent standardization efforts by OMERACT [15,19]. Current EULAR recommendations similarly focus on specific organ strategies; however, this approach may inadequately guide clinicians, who are required to manage the complex, multi-organ nature of SSc in routine clinical practice.

Another key limitation in the literature is the scarcity of studies directly comparing different biologic agents in SSc patients and, more broadly, describing how they are used across distinct clinical phenotypes in real-life settings. Two retrospective analyses conducted on the EUSTAR cohort compared 93 SSc patients treated with TCZ with large SSc cohorts treated with other immunosuppressive therapies (including cyclophosphamide, methotrexate, azathioprine, mycophenolate mofetil, abatacept, and rituximab): both studies failed to demonstrate significant differences in FVC trends between treatment groups during a median follow-up time of approximately 12 months [12,20]. To date, only one study has directly and exclusively compared TCZ and RTX: Goldman et al.’s retrospective analysis of 127 real-world SSc-ILD patients, which examined both agents with or without background mycophenolate mofetil and showed comparable efficacy on FVC stabilization over 24 months. However, this study focused exclusively on lung function outcomes without evaluating other disease domains [11].

Our study adds to this body of evidence by providing observational data on the use of TCZ and RTX across multiple disease domains in a real-world SSc cohort. In our center, TCZ was mainly used as a later line or rescue therapy, often in patients with more longstanding disease, lower baseline mRSS and more impaired DLCO%, whereas RTX was more frequently introduced earlier in the disease course, generally in patients with higher mRSS. Accordingly, although baseline global disease activity did not significantly differ between groups, the two cohorts reflected distinct treatment indications. In this context, both TCZ and RTX were associated with a significant reduction in mRSS, supporting their potential efficacy on skin involvement in real-life SSc patients. The more pronounced mRSS improvement observed in RTX-treated patients is likely influenced by higher baseline skin scores and earlier use in the treatment sequence, which confer a greater potential for measurable change.

In order to assess overall disease activity, independently of the main involved domain, and, thus, to overcome the previously mentioned baseline differences, we calculated the RAI—a composite disease activity score—at each time point. This score, although not independent of mRSS, DLCO and CRP, still provides a valuable indicator of global disease activity. Within our cohort, both treatment groups demonstrated a rapid and significant RAI reduction. The RTX group showed a slightly higher rate of clinical remission at T6, which may reflect the pronounced mRSS improvement observed in these patients. Additionally, CRP levels declined throughout the follow-up period in both groups. This reduction in CRP—a marker of systemic inflammation and established negative prognostic indicator in SSc—further supports the anti-inflammatory efficacy of both treatments [21]. These comparisons must be interpreted with caution, given some confounders that may influence changes in mRSS, CRP, and RAI after treatment compared with baseline in the two groups (ex. age, sex, baseline mRSS).

Regarding lung progression, our findings were discordant: over a 24-month period both TCZ- and RTX-treated patients demonstrated a significant decline in FVC%, but preserved DLCO% values. Even if the analysis was adjusted for disease duration, however, these results should be interpreted with caution, as the two SSc-ILD cohorts—though similar in baseline FVC%—still differed in baseline DLCO% (*p*: 0.024). Moreover, when applying the OMERACT definition of lung progression, a low number of events—without between-group differences—was observed. Overall, these data point to a modest decline in FVC%, relative preservation of DLCO% and a low rate of functional progression in both treatment cohorts, but they do not allow firm conclusions on any differential effect of TCZ versus RTX on lung function, given the small sample size and baseline imbalances. Of note, dissociation of FVC% and DLCO% trends observed in our study could mirror evidence from the literature. Indeed, both for RTX and TCZ trials, over time changes in DLCO% were modest and heterogeneous compared to FVC%. Though both these spirometric parameters are established predictors of mortality in SSc patients, they reflect different pathophysiological pathways, FVC% being driven mostly by restrictive ventilatory impairment, while DLCO% by both interstitial abnormalities and pulmonary vascular dysfunction [2]. Consequently, DLCO may require longer treatment duration and can be influenced by concomitant vasoactive treatments. However, the relative preservation of DLCO% observed in FocuSSced and DESIRES trials suggests that immunosuppressive treatment may contribute to attenuate downstream vascular–interstitial damage over time [4,7]. Further research is warranted to better characterize DLCO% trends in systemic sclerosis by analyzing separately patients with group I pulmonary hypertension (PH) and those with interstitial lung disease, with or without concomitant ILD-associated PH [22].

Finally, our analysis confirms the overall favorable safety profiles of both TCZ and RTX over a 24-month follow-up period, longer than most published trials on this topic, consistent with findings from other rheumatic diseases and from real-life SSc patients [23,24]. Indeed, no patients discontinued either drug due to adverse events and overall only one death and one drug failure were reported. Nonetheless, the limited duration of follow-up precludes definitive conclusions regarding the long-term safety and retention rate of TCZ in this group of patients.

Overall, our retrospective observational data reflect a personalized, phenotype-oriented use of TCZ and RTX in systemic sclerosis, where treatment selection is tailored according to disease stage, dominant organ involvement, baseline activity, and clinical phenotype rather than applied uniformly across patients. In this context, our study was primarily designed to describe real-world patterns of biologic use and associated clinical trajectories across heterogeneous SSc phenotypes, rather than to provide a head-to-head efficacy comparison between the two agents. Some important limitations need to be underlined, including the monocentric and retrospective design of the study and the limited sample size, which precluded further subgroup analyses or more extensively adjusted models (ex. age, comorbidities). Two different physicians were responsible for mRSS assessment—although both expert rheumatologists involved in our Scleroderma Unit—and PFTs were not performed by a unique examiner: these factors could have affected the reliability of our results. As previously discussed, baseline imbalances in disease duration, baseline DLCO% and antifibrotic treatment, combined with heterogeneity in therapeutic indications and potential residual confounding by indication, may hamper the validity of our findings. Radiologic measures of interstitial lung disease extent, such as chest CT-based assessment, were not recorded. In addition, no matching or propensity-based methods were applied, and treatment allocation was entirely driven by clinical judgment, which further limits the interpretability of between-group comparisons and prevents any definitive inference on comparative effectiveness between TCZ and RTX. Lastly, six TCZ-treated patients did not complete the full 24-month follow-up: although all had received at least 12 months of treatment (except for the patient who died during the observational period), this may have influenced the study results.

## 5. Conclusions

This observational study focused on TCZ and RTX in real-world SSc patients, evaluating both global clinical activity and lung function over 24 months. Our findings suggest that both treatments are associated with a rapid reduction in disease activity and inflammatory markers, and with a low rate of lung functional progression, thereby supporting their therapeutic value in the management of SSc, including patients with complex and heterogeneous phenotypes. The encouraging clinical outcomes observed with TCZ, particularly in patients with more advanced disease and lower baseline mRSS, warrant additional investigation in larger, prospectively designed studies. Given the substantial baseline imbalances and confounding by indication, our data do not allow firm conclusions on the comparative effectiveness of TCZ versus RTX but rather support their use in different clinical scenarios. Future studies with larger cohorts, standardized definitions of progression and more precise phenotyping will be essential for refining treatment selection criteria and improving outcomes in this complex disease.

## Figures and Tables

**Table 1:** Baseline characteristics of patients.

	TCZ (27)	RTX (23)	*p*-Values
Female, *n* (%)	25 (92.59)	21 (91.30)	1.000
Caucasian, *n* (%)	21 (77.77)	21 (91.30)	0.479
Age, years, mean (SD)	49.37 (14.49)	48.30 (8.49)	0.785
Disease duration, months, median (IQR)	98 (95.50)	37 (20.50)	<0.001
dcSSc, *n* (%)	23 (85.18)	23 (100.00)	0.115
ATA, *n* (%)	23 (85.18)	19 (82.61)	1.000
ACA, *n* (%)	0 (0)	1 (4.30)	0.460
ILD, *n* (%)	19 (70.37)	18 (78.26)	0.747
PH, *n* (%)	2 (7.41)	3 (13.04)	0.868
RA overlap, *n* (%)	2 (7.41)	2 (8.69)	1.000
FVC%, mean (SD) *	73.68 (18.78)	81.16 (20.66)	0.320
DLCO%, mean (SD) *	41 (24.00)	56 (20.91)	0.024
Arthritis, *n* (%)	15 (55.55)	9 (39.13)	0.272
Myocarditis, *n* (%)	4 (14.81)	3 (13.04)	1.000
Steroid, *n* (%)	25 (92.60)	21 (91.30)	1.000
Steroid dosage, mg, median (IQR)	7.50 (5.00)	10.00 (5.00)	0.572
MTX, *n* (%)	11 (40.74)	12 (52.17)	0.570
MMF, *n* (%)	5 (18.52)	7 (30.43)	0.508
NNT, *n* (%)	7 (25.92)	0 (0.00)	0.011
mRSS, median (IQR)	3 (8.00)	14 (10.00)	<0.001
CRP, mg/dL, median (IQR)	0.50 (8.00)	0.30 (1.25)	0.168
RAI > 2.5, *n* (%)	21 (77.78)	21 (91.30)	0.147
Initiated for ILD, *n* (%)	15 (55.55)	10 (43.48)	0.571
Initiated for skin disease, *n* (%)	3 (11.11)	13 (56.52)	0.008
Initiated for myocarditis, *n* (%)	3 (11.11)	0 (0.00)	0.240
Initiated for arthritis, *n* (%)	6 (22.22)	0 (0.00)	0.005

ACA: anti-centromere antibody, ATA: anti-topoisomerase I antibody, CRP: C-reactive protein, dcSSc: diffuse cutaneous systemic sclerosis, DLCO%: diffusion capacity for carbon monoxide, FVC%: forced vital capacity, ILD: interstitial lung disease, mRSS: modified Rodnan Skin Score, MTX: methotrexate, NNT: nintedanib, PH: pulmonary hypertension, RA: rheumatoid arthritis, RAI: revised EUSTAR activity index 2017. Fisher test for categorical variables; Mann–Whitney U test and T-test for continuous variables, * SSc-ILD cohorts: TCZ *n* = 19, RTX *n* = 18.

**Table 2:** Over time changes in clinical parameters during the observational period in the two groups.

	T6 vs. T0		T12 vs. T0		T24 vs. T0		T12 vs. T6		T24 vs. T12		T6 vs. T0	
Tocilizumab	Delta	*p*	Delta	*p*	Delta	*p*	Delta	*p*	Delta	*p*	Delta	*p*
mRSS	−1.000	0.010	−1.000	0.011	−1.000	0.001	0.000	0.044	0.000	<0.001	−1.000	0.011
CRP	−0.500	0.009	−0.500	<0.001	−0.500	<0.001	0.000	0.036	0.000	0.150	−0.500	<0.001
RAI	−1.572	<0.001	−2.664	<0.001	−2.832	<0.001	−1.092	0.007	−0.168	0.006	−2.664	<0.001
Rituximab	Delta	*p*	Delta	*p*	Delta	*p*	Delta	*p*	Delta	*p*	Delta	*p*
mRSS	−6.000	<0.001	−8.000	<0.001	−10.00	<0.001	−2.000	0.003	−2.000	0.050	−6.000	<0.001
CRP	−0.300	0.003	−0.300	<0.001	−0.100	0.090	0.000	0.125	0.200	0.010	−0.300	0.003
RAI	−3.624	<0.001	−3.750	<0.001	−3.582	<0.001	−0.126	0.080	0.168	0.067	−3.624	<0.001

CRP: C-reactive protein, mRSS: modified Rodnan Skin Score, RAI: revised EUSTAR activity index 2017. Analysis of RAI conducted on patients scoring ≥ 2.5 at T0 (TCZ *n* = 21, RTX *n* = 21). Wilcoxon signed-rank test with respective Delta (change) and *p*-value with Bonferroni correction.

## Data Availability

The datasets used and/or analyzed during the current study are available from the corresponding author on reasonable request.

## Supplementary Materials

The following supporting information can be downloaded at https://www.mdpi.com/article/10.3390/jpm16040186/s1, Table S1: Additional baseline characteristics of the cohorts; Table S2: Comparison between over time changes in clinical parameters in the two groups during the observational period.

## Abbreviations

The following abbreviations are used in this manuscript:

ACA	Anti-centromere antibody
AHSCT	Autologous hematopoietic stem cell transplantation
ATA	Anti-topoisomerase I antibody
CRP	C-reactive protein
CT	Computed tomography
CTD	Connective tissue disease
CTD-ILD	Connective tissue disease-associated interstitial lung disease
CYC	Cyclophosphamide
dcSSc	Diffuse cutaneous systemic sclerosis
DLCO%	Diffusing capacity for carbon monoxide (percentage of predicted)
FDA	Food and Drug Administration
FVC%	Forced vital capacity (percentage of predicted)
IL6	Interleukin-6
ILD	Interstitial lung disease
IS	Immunosuppressants
MMF	Mycophenolate mofetil
mRSS	Modified Rodnan Skin Score
MTX	Methotrexate
NNT	Nintedanib
NSIP	Non-specific interstitial pneumonia
PFT	Pulmonary function test
PH	Pulmonary hypertension
RAI	Revised EUSTAR Activity Index 2017
RTX	Rituximab
SSc	Systemic sclerosis
TCZ	Tocilizumab
