# Supplementary material for: Tocilizumab and Rituximab in Systemic Sclerosis: A Real-Life Retrospective Observational Study Across Different Clinical Phenotypes
Source: J Pers Med. 2026 Mar 30;16(4):186. doi: 10.3390/jpm16040186 (PMC13117874; doi:10.3390/jpm16040186)
Supplement: Supplementary file 1 [file jpm-16-00186-s001.zip › jpm-4185496-supplementary.pdf]

Table S1. Additional baseline characteristics of the cohorts. ANA: anti-nuclear antibodies, anti-RNP: anti-U1 ribonucleoprotein, anti-SSA: anti-Sjogren Syndrome A, ARA: anti-RNA polymerase III, DU: digital ulcers, GI: gastrointestinal tract.

	**TCZ (27)**	**RTX (23)**	**p-values**
Disease onset age, mean (SD)	41.26 (16.06)	45.83 (8.69)	0.110
Smokers, n (%)	2 (7.41)	7 (30.43)	0.062
ANA, n (%)	27 (100.00)	23 (100.00)	1.000
ARA, n (%)	1 (3.70)	2 (8.69)	0.588
anti-RNP, n (%)	1 (3.70)	0 (0.00)	1.000
anti-SSA, n (%)	5 (18.52)	1 (4.35)	0.199
DU, n (%)	17 (62.96)	16 (69.56)	0.459
Myositis, n (%)	2 (7.41)	1 (4.35)	1.000
Upper GI, n (%)	16 (59.26)	17 (73.91)	0.372
Lower GI, n (%)	2 (7.41)	2 (8.69)	0.470

Table S2. Comparison between overtime changes of clinical parameters in the two groups during the observational period. CRP: C reactive protein, mRSS: modified Rodnan Skin Score, RAI: revised EUSTAR activity index 2017. Analysis of RAI conducted on patients scoring ≥ 2.5 at T0 (TCZ n=21, RTX n=21). Mann-Whitney U test with respective p-value with Bonferroni correction.

	**Timepoint**	**TCZ Δ median (IQR)**	**RTX Δ median (IQR)**	**p-value**
mRSS	T6 vs T0	-1.00 (2.00)	-6.00 (6.00)	< 0.001
mRSS	T12 vs T0	-1.00 (2.00)	-8.00 (6.50)	< 0.001
mRSS	T24 vs T0	-1.00 (2.50)	-10.00 (7.50)	< 0.001
CRP	T6 vs T0	-0.50 (3.95)	-0.30 (0.80)	0.944
CRP	T12 vs T0	-0.50 (6.5)	-0.30 (0.45)	0.291
CRP	T24 vs T0	-0.50 (6.5)	-0.10 (0.77)	0.009
RAI	T6 vs T0	-1.57 (1.12)	-3.62 (2.17)	< 0.001
RAI	T12 vs T0	-2.66 (2.25)	-3.75 (2.59)	0.062
RAI	T24 vs T0	-2.83 (2.25)	-3.58 (2.93)	0.992
